# Thermal Behavior of Pinan-2-ol and Linalool

**DOI:** 10.3390/molecules18078358

**Published:** 2013-07-16

**Authors:** Janne Leiner, Achim Stolle, Bernd Ondruschka, Thomas Netscher, Werner Bonrath

**Affiliations:** 1 Institute for Technical Chemistry and Environmental Chemistry (ITUC), Friedrich-Schiller University Jena, Lessingstr. 12, D-07743 Jena, Germany; 2 Research and Development, DSM Nutritional Products, P.O. Box 2676, CH-4002 Basel, Switzerland

**Keywords:** biradicals, cyclobutane fragmentation, isomerization, pericyclic reactions, pyrolysis, terpenoids, thermal rearrangement

## Abstract

Linalool is an important intermediate for syntheses of isoprenoid fragrance compounds and vitamins A and E. One process option for its production is the thermal gas-phase isomerization of *cis*- and *trans*-pinan-2-ol. Investigations of this reaction were performed in a flow-type apparatus in a temperature range from 350–600 °C and a residence time range of 0.6–0.8 s. Rearrangement of the bicyclic alcohol led to linalool, plinols arising from consecutive reactions of linalool and other side products. Effects of residence time, temperature, surface-to-volume-ratio, carrier gas, and the presence of additives on yield and selectivity were studied. Furthermore, the effects of such parameters on ene-cyclization of linalool affording plinols were investigated. Results indicate that manipulation of the reaction in order to affect selectivity is difficult due to the large free path length to other molecules in the gas phase. However, conditions have been identified allowing one to increase the selectivity and the yield of linalool throughout pyrolysis of pinan-2-ol.

## 1. Introduction

Terpenoids are a large class of natural occurring compounds. Besides being important in Nature, for example as pheromones or antioxidants, they are valuable fine chemicals used in the fragrance, flavor, and pharmaceutical industry [1,2]. Terpenoids are oligomers or polymers of isoprene. Moreover they offer an access to complex molecular structures and in some cases they are part of Nature’s pool of chiral compounds. Those facts make them interesting starting materials for the synthesis of chiral ligands or auxiliaries used in enantioselective catalytic syntheses [3,4,5]. In this context, rearrangement reactions of monoterpenoids play a major role in the industrial synthesis of fine chemicals, flavors, and fragrances. For instance, the gas-phase isomerization of the monoterpenoid pinan-2-ol (**1**) results in the formation of the acyclic allylic alcohol linalool (**2**) [6,7].

**Scheme 1:**
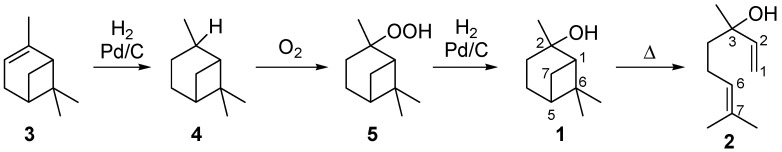
Synthesis of pinan-2-ol (**1**) starting from α-pinene (**3**).

Linalool, (*S*)- or (*R*)-3,7-dimethylocta-1,6-dien-3-ol, can be used as a key intermediate in the synthesis of vitamins A and E. Furthermore, it serves as important reagent in the syntheses of flavor and fragrance compounds, which are used in perfumes, cosmetics, or household cleaning agents [7,8]. A possible synthetic route to receive **2** is based on the reaction of 6-methyl-5-hepten-2-one by base-catalyzed ethynylation to dehydrolinalool followed by selective hydrogenation of the triple bond to a double bond in the presence of a Pd/carbon catalyst [6,9]. Several synthetic possibilities are outlined in the literature to obtain **2** from natural sources [10,11,12]. Beside isolation from essential oils, α-pinene and β-pinene are among the preferred starting materials, as they are rather cheap products available from crude sulfate turpentine (CST), a by-product from the pulp and paper industry [1,13]. The corresponding four step synthesis starts with the Pd-catalyzed reduction of α-pinene (**3**) which leads to *cis*- and *trans*-pinane (**4**) (Scheme 1). The subsequent oxidation affords *cis*- and *trans*-pinan-2-hydroperoxide (**5**) which is reduced over Pd/carbon catalysts to *cis*- and *trans*-**1** [14,15,16]. The last step is the pyrolysis of **1** to **2** with the by- and side-products mentioned below [9,13,14].

**Scheme 2:**
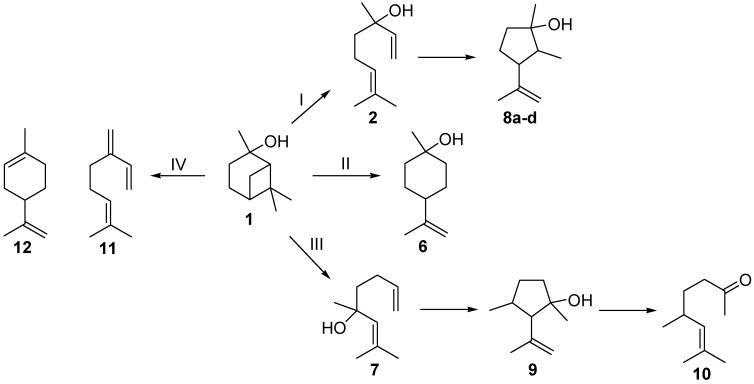
Simplified reaction scheme for the isomerization of pinan-2-ol (**1**).

The last step of the reaction sequence outlined in Scheme 1 is another commonly used method for the production of **2**. The gas-phase rearrangement of **1** in the temperature range of 450–600 °C leads to the formation of acyclic **2** as the main product but also to further side- and by-products (Scheme 2) [17]. Ohloff and Klein identified several cyclopentanols, namely plinols **8a**–**d** (pathway I), which result from a consecutive reaction of **2** [17,18,19]. They also identified two by-products, namely the monocyclic *p*-menthane-type compound β-terpineol (**6**, pathway II) and 5,7-dimethyloct-6-ene-2-one (**10**), a methyl ketone whose structure was elucidated by Coxon and co-workers [20]. Vandewiele *et al.* reported that **10** was formed through a subsequent reaction of acyclic isolinalool (**7**) and the corresponding cyclopentenol derivative **9** (pathway III) [19,21]. In addition Semikolov *et al.* reported the presence of hydrocarbons myrcene (**11**) and limonene (**12**) originating from dehydration reactions of **2** and **6**, respectively [22].

Small variations of reaction conditions such as temperature, reactor geometry, *etc.*, result in differences in linalool selectivity and yield. Side reactions can also be influenced or even suppressed by using short reaction times or additives [14,15,22]. For example, Semikolenov *et al.* added small amounts of pyridine to a solution of pinan-2-ol to suppress side reactions and increase linalool selectivity [22]. Contrarily, Buddoo *et al.* preformed the isomerization reaction in a multichannel micro-reactor system to increase conversion and selectivity compared with traditionally used tubular reactor systems [23].

The current paper presents our results concerning the thermal behavior of pinan-2-ol (**1**) and its main product linalool (**2**). Reactions were carried out in a flow-type reactor within a temperature range of 350–600 °C using oxygen-free N_2_ as carrier gas. The aim of this work was to investigate the influence of several reaction parameters like on the selectivity and yield of linalool formation in the process of pinane-2-ol isomerization. Parameters like residence time, temperature, surface-to-volume-ratio, carrier gas, and additives were assessed.

## 2. Results and Discussion

**Figure 1:**
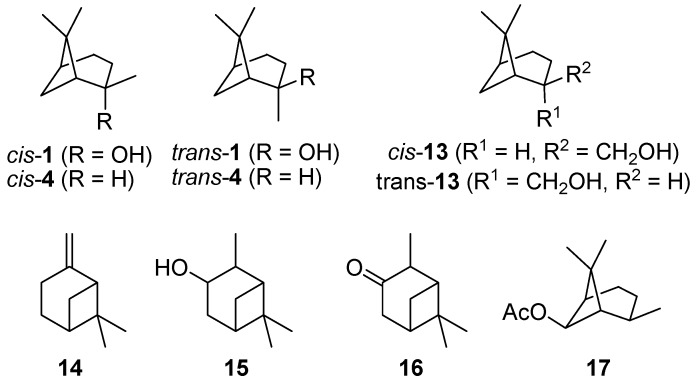
Some important structures and further bicyclic monoterpenes.

Commercially available *cis*-pinan-2-ol (*cis*-**1**; 85–90 wt% *cis*) was used in the pyrolysis experiments. For comparison, samples of *trans-*pinan-2-ol (*trans*-**1**; 81–89 wt% *trans*) were prepared by synthesis and subsequent chromatographic separation of *cis*/*trans* mixtures according to literature procedures [14,15,16,24], as depicted in Scheme 1. The structures of some bicyclic terpenes are shown in Figure 1.

### 2.1. Thermal Behavior of Pinan-2-ol

**Figure 2:**
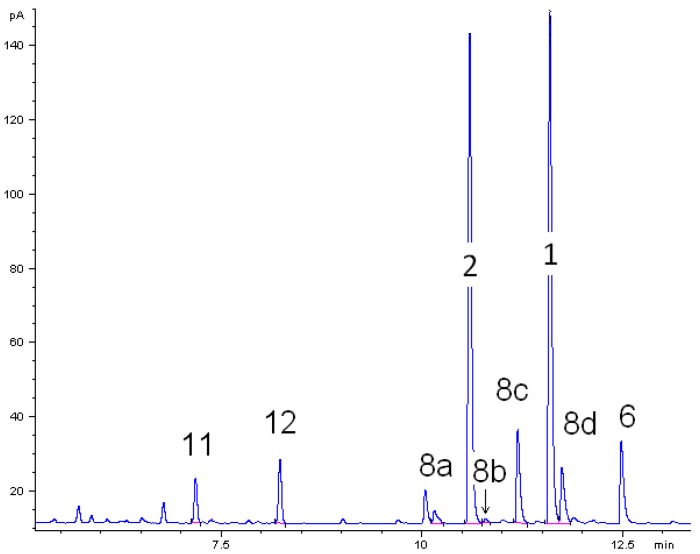
Gas chromatogram (GC-FID) of the liquid pyrolysis products of *cis*-pinan-2-ol (**1**) thermal isomerization. Reaction conditions: 30 µL **1**, carrier gas: N_2_, flow rate: 0.8 L min^−1^, solvent for dissolution of products: ethyl acetate.

The thermal behavior of monoterpenoids **1** and **2** were studied. For both components the experimental conditions were the same in each individual isomerization experiments. A gas chromatogram of the liquid product mixture in ethyl acetate obtained from the thermal isomerization of *cis*-**1** at 500 °C is depicted in Figure 2. Beside target product linalool (**2**), consecutive reaction products of **2** plinols **8a**–**d**, β-terpineol (**6**) from parallel isomerization reaction channel of **1**, and dehydration products myrcene (**11**) and limonene (**12**) were identified. Peaks which are not assigned in Figure 2 originate from impurities of the starting material like borneol. The absolute and relative concentration of those impurities remained constant throughout all experiments. Thus, formation as a result of the experimental conditions can be ruled out.

**Figure 3:**
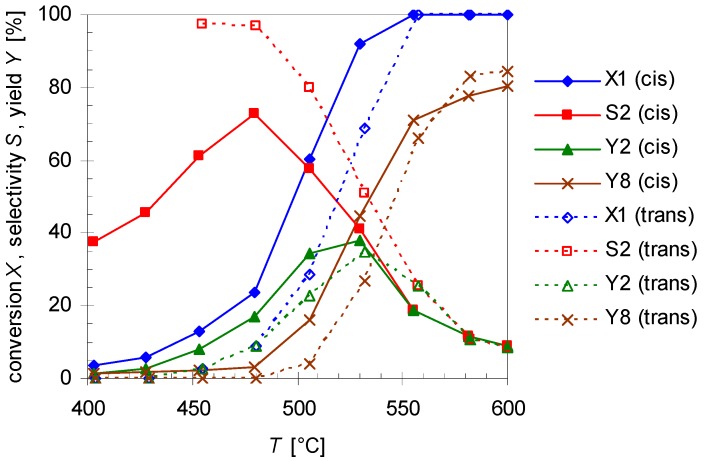
Temperature dependency of pinan-2-ol conversion *X***_1_** (diamonds), linalool selectivity *S***_2_** (squares), linalool yield *Y***_2_** (triangles), and plinol yield *Y***_8_** (crosses) for the pyrolysis of *cis*- (solid line) and *trans*-**1** (dotted lines). Reaction conditions: 30 µL **1**, carrier gas: N_2_, flow rate: 0.8 L min^−1^, *τ*: 0.6–0.8 s.

The dependency of *cis*- and *trans*-pinan-2-ol conversion (*X***_1_**) from the reaction temperature (*T*; 400–600 °C) with a flow rate of 0.8 L min^-1^ N_2_ was studied and results are depicted in Figure 3. Conversion were calculated according to Equation (1), wherein *i* denotes compound **1** (Figure 3) or **2** (Section 2.2):

(1) $$X_{i}=1-\frac{\left[i\right]}{100}$$

The diagram reveals that the conversion of both isomers increases with *T*, but it seems that *cis*-**1** is more reactive than the *trans*-isomer. Conversion of *cis*-**1** starts at 400 °C which is 50 K below the onset temperature for *trans*-**1**. This is also the explanation for the fact that the selectivity curve (*S***_2_**) for the *trans*-isomer starts at 450 rather than 400 °C. However full conversion for isomers is reached at 550 °C. Contrarily to pyrolysis of **3**, leading to racemization at C(2) by [1,3]C shift [25,26], no interconversion of the single stereoisomer was observed in the present case, similar to the behavior of *cis*- and *trans*-**4** [27].

Selectivity *S*_i_ and yield *Y*_i_ for products were calculated according to Equations (2) and (3), respectively, wherein *i* resembles an individual product or the summarized values for a specific reaction pathway according to Scheme 2:

(2) $$S_{i}=\frac{Y_{i}}{X_{1}}=\frac{\left[i\right]}{X_{1}}$$

(3) $$Y_{i}=\left[i\right]$$

The main product of the thermal isomerization of *cis-***1** is **2** with a maximal selectivity *S***_2_** of 73% (Figure 3). Again the isomers show different results considering yield *Y***_2_** and selectivity of **2**. At equal temperatures the selectivity curve from experiments with *cis*-**1** has lower values than that of the *trans*-alcohol but both curves decreased to the same final value. Also both isomers show the same profile for *Y***_2_**, reaching a maximum and decrease afterwards, but while the curve shape is the same the maxima differ. The maxima of *Y***_2_** is shifted for experiments with *cis*-**1** to 475 °C which is 25 K lower then observed for *trans*-**1**. Disadvantage of the reaction is that formed **2** underwent consecutive reactions starting at ca. 450 °C leading to **8**. This resulted in lower selectivity for **2**, whereas the yield of **8** (*Y***_8_**) increases (Figure 3). Although four different plinols were formed, which appear as four separate peaks in the chromatogram (Figure 2), they are summarized and treated as one compound in first approximation. Compound **2** which is generated from *cis*-**1** as well as pyrolysis experiments with **2** revealed a higher reactivity regarding the transformation into **8** compared to the reaction of the *trans*-isomer. In both cases **8** was formed at 400 °C, whereas in case of *trans*-**1** the formation of **8** starts at 475 °C. Similar behavior has been reported by Ohloff and Klein [17] and is also known from the pyrolysis of *cis*- and *trans*-**4**, β-citronellene, and isocitronellene as parent hydrocarbon compounds for **1**, **2**, and **7**, respectively [27,28], as well as for the isomerization of *cis*- and *trans*-myrtanol (**13**; Figure 1) [29].

Beside products **2** and **8**, other compounds were formed during the thermal isomerization of **1**: β-terpineol (**6**), myrcene (**11**), and limonene (**12**) could be identified but were formed in low amounts only (see Figure 2). Products from reaction pathway III as indicated in Scheme 2 were not observed. Therefore the reaction products can be classified according to the following main reaction pathways starting from **1**.

i) Pathway I (index p2) leads to **2** and **8**.
ii) Pathway II (index p3) affords cyclohexanol **6**.
iii) Pathway IV (index p4) summarizes dehydration products **11** and **12**.

**Figure 4:**
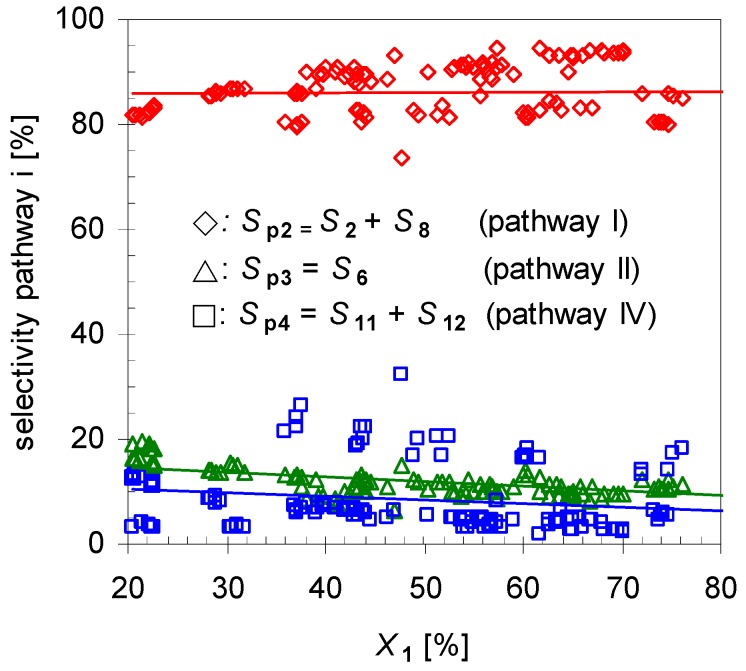
Dependency of overall linalool selectivity (S**_p2_**; diamonds), β-terpineol selectivity (*S***_p3_**, triangles) and hydrocarbons (*S***_p4_**, squares; pathways according to Scheme 2) from conversion of pinan-2-ol (*X***_1_**). Reaction conditions: 30 µL **1**, carrier gas: N_2_, flow rate: 0.4–1.2 L min^−1^, *τ*: 0.6–0.8 s.

Reaction analysis with respect to selectivity for each pathway *S*_pi_ by plotting the dependency of *S*_pi_ to *X***_1_** (Figure 4) indicates that *S*_p2_ is the main route accounting for ≥80% of the formed products. The remaining two pathways have a minor selectivity with summarized values below 20%, whereas the difference between those is low. To increase the yield of **2** one option is the suppression of those minor pathways, which will be investigated later on.

**Figure 5:**
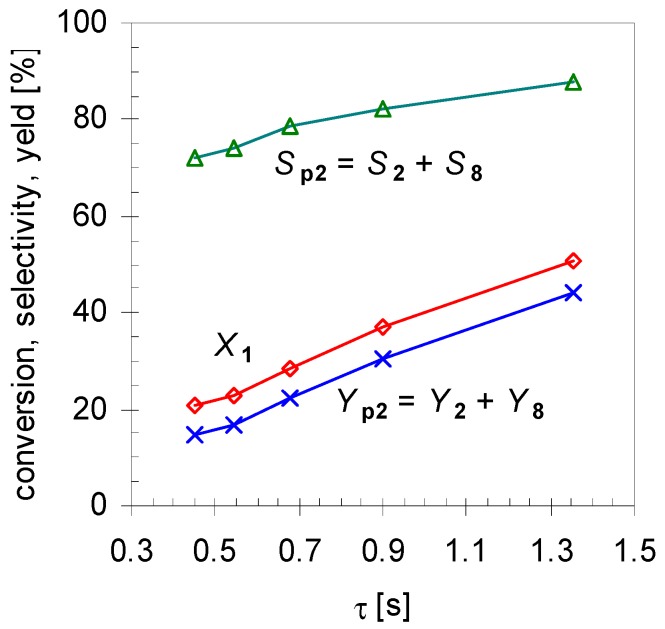
Effect of residence time *τ* on conversion (*X***_1_**; diamonds), overall selectivity (S**_p2_**; triangles) and yield for products from pathway I (*Y***_p2_**; crosses; see Scheme 2). Reaction conditions: 30 µL **1**, carrier gas: N_2_, flow rate: 0.4–1.2 L min^−1^, *T*: 480 °C.

Furthermore, experiments revealed that the initial formation is basically independent of *X***_1_** in the range of 20 < *X***_1_** < 80%. Conversions lower than 20% were not considered in calculating the mean values because these resulted in signals below the detection limit of the GC-FID for some minor products. On the other hand, for conversions higher than 80% **2** started to isomerize, forming products **8a**–**d**, and these values were not included for averaging either. Comparison of these results with those of the structurally related monoterpene β-pinene (**14**), studied by Stolle *et al.*, shows similar effects [30]. Like **1** gas-phase isomerization of **14** offers three reaction pathways with one preferred way which led to the formation of acyclic **11** and its consecutive products [19,31]. Figure 5 shows the dependency of the product composition for pathway I on the average residence time *τ* at a constant reaction temperature (480 °C). With increasing *τ* the values for *X***_1_**, *S*_p**2**_, and *Y*_p**2**_ increase linearly, which indicates that the reaction follows a first-order kinetic. It is difficult however to compare these results with other published results [14,15,22], because pyrolysis apparatus always differ.

**Figure 6:**
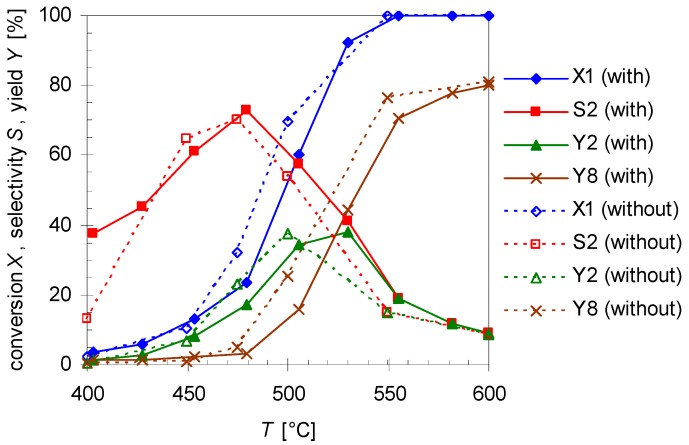
Temperature dependency of pinan-2-ol conversion *X***_1_** (diamonds), linalool selectivity *S***_2_** (squares), linalool yield *Y***_2_** (triangles), and plinol yield *Y***_8_** (crosses) for the pyrolysis of **1** with (solid line) and without insert (dotted lines; cf. Table 1). Reaction conditions: 30 µL **1**, carrier gas: N_2_, flow rate: 0.8 L min^−1^, *τ*: 0.6–0.8 s.

To study the influence of surface-to-volume-ratio (*S/V*) on the reactivity for the isomerization of **1** experiments with varied reactor volume *V*_R_ (Table 1) and constant flow rate of 0.8 L min^−^^1^ N_2_ were carried out. Therefore, pyrolysis experiments with a quartz insert (solid lines; reactor geometry = hollow cylinder) [30] and without insert (dotted lines; reactor geometry = cylinder) were undertaken. Results are shown in Figure 6.

As presented in Figure 6, the thermal behavior of **1** is very similar with and without insert, while selectivity and yield of the products differ slightly. The conversion without insert is insignificantly higher at equal temperatures but otherwise has the same curve shape and the same conversion range like for experiments with the insert (which has been used in previous experiments). Therefore, *S/V* has presumably no influence on the reaction, contrarily to the effect of the average residence time τ and the reaction temperature *T*. As the reactions have been carried out at the same flow rate, but at different reactor volumes (Table 1) the values for *τ* are lower for experiments with the insert (*τ* = 0.6–0.8 s) than for those in its absence (*τ* = 0.9–1.2 s). Assuming isobaric conditions, average residence time *τ* was calculated using Equation (4):

(4) $$\tau ≅\frac{V_{R}}{\left(V_{E}^{*}+V_{N2}^{*}\right)\cdot \frac{T_{R}}{T_{rt}}}$$

wherein *V*_R_, V*_E_, V*_N2_, *T*_R_, and *T*_rt_ denote the reactor volume, the volumetric flow rate of the substrate, the volumetric flow rate of the carrier gas N_2_, the reactor temperature, and the room temperature, respectively. For **2** both curves (*S***_2_** and *Y***_2_**; Figure 6) have a similar profile each passing through a maximum, but with insert slightly higher values can be observed for the whole selectivity curve and its maximum. A similar behavior has been observed for the pyrolysis of **14** [30]. It is likely that terpenes and terpenoids behave similar [32,33], especially since the same pyrolysis apparatus was used.

**Figure 7:**
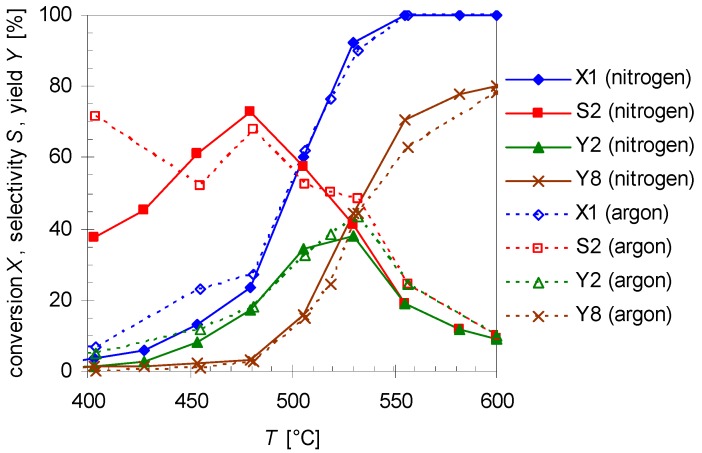
Temperature dependency of pinan-2-ol conversion *X***_1_** (diamonds), linalool selectivity *S***_2_** (squares), linalool yield *Y***_2_** (triangles), and plinol yield *Y***_8_** (crosses) for the pyrolysis of **1** with nitrogen (solid line) and argon (dotted lines) as carrier gas. Reaction conditions: 30 µL **1**, flow rate: 0.8 L min^-1^, *τ*: 0.6–0.8 s.

Experiments with argon as another carrier gas were carried out as well using the similar experimental condition as before. Figure 7 compares the results for pyrolysis with different carrier gases at various reaction temperatures (*T*; 400–600 °C) with a flow rate of 0.8 L min^−1^. As results show the thermal behavior of **1** and its products do not improve significantly. Furthermore, the same by- and side-products are found. Although with argon as carrier gas the conversion of **1** (*X***_1_**) and selectivity (*S***_2_**) and yield (*Y***_2_**) of **2** are a slightly higher at the lower temperatures, above 475 °C the values are nearly the same. Hence, variation of the carrier gas has presumably no effect on the reaction.

### 2.2. Thermal Behavior of Linalool

**Scheme 3:**
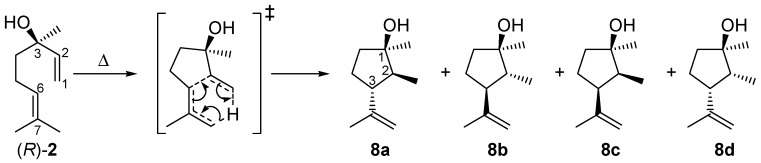
Isomerization of (*R*)-linalool (**2**) to plinols **8a**–**d**.

**Figure 8:**
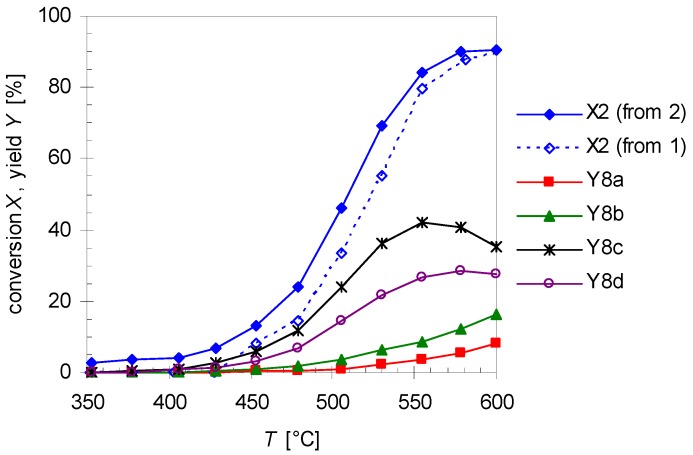
Temperature dependency of the linalool conversion *X_2_* extracted from pyrolysis experiments with **1** (dotted line, open diamonds) and from experiments with **2** (solid line, closed diamonds) and the yields of plinols **8a**–**d** (*Y***_8i_**) from pyrolysis experiments with linalool **2**. Reaction conditions: 30 µL starting material, carrier gas N_2_, flow rate: 0.8 L min^−1^, *τ*: 0.6–0.8 s.

As shown in Figure 3, the yield of **2** drops with increasing temperature after passing a maximum due to consecutive rearrangement to plinols **8a**–**d** (Scheme 3). The reaction scheme explaining the formation of **8** from **2** (the mechanistic details of the reaction will be discussed in Section 2.4) is based on the fact that optical pure (*R*)-**2** is used as starting material for the following experiments. With this prerequisite it is possible to assign the absolute configuration of the carbon atoms in compounds **8a**–**d**. To study the formation of these products, conversion of **2** (*X***_2_**) was analyzed under the same conditions as the thermal isomerization of **1**. Therefore, Figure 8 shows the high agreement in progression of *X***_2_** from pyrolysis experiments with **2** and *X***_2_** extracted from the experimental data concerning pyrolysis of **1**. Previous studies with myrcene and ocimene generated from α-pinene (**3**) [26] and β-pinene (14) [30], respectively, showed that this behavior is typical for the ring opening of bicyclic monoterpenes to the corresponding acyclic products [19,33].

In Figure 8 the increase in the yield of the four rearranged products (*Y***_8i_**) is presented. Designation of the relative orientation of the substituents in **8a**–**d** derives from previous reports according to their retention times whereby **8a** has the lowest one and **8d** elutes last on a HP5 GC-column (Scheme 3 and Figure 2) [17,18]. For assignment of the absolute configuration see the previous Section. Based on the assumption that the asymmetric carbon center in **2** is not changed during pyrolysis the absolute configuration of this carbon atom in **8** can be determined and the absolute configuration of the remaining atoms can be ascribed taking into account experiments from the literature. The four products build up in different yields at equal temperatures with **8c** having the highest yield and **8a** the lowest, resulting in the following sequence: *Y***_8c_** > *Y***_8d_** > *Y***_8b_** > *Y***_8a_**. This order is in accordance with previously published results using a column with similar polarity [17,18,20]. It is also evident that at about 550 °C *Y***_8c_** passes through a maximum as does *Y***_8d_** at 590 °C forming yet unknown species, probably decomposition products since there are multiple peaks at shorter retention times. There have also been experiments changing residence time and like previous experiments with **1**, product yield increases with increasing *τ*. These results are in agreement with results from previous studies although different parameters have been set up [22]. The product distribution reported in Figure 8 is independent of the type of pyrolysis experiment. Thermal isomerization of **2** as well as consecutive reactions of **2** in the gas-phase rearrangement of **1** led to the same products with similar distribution.

### 2.3. Mechanism of the Rearrangement of Pinan-2-ol into Linalool

**Scheme 4:**
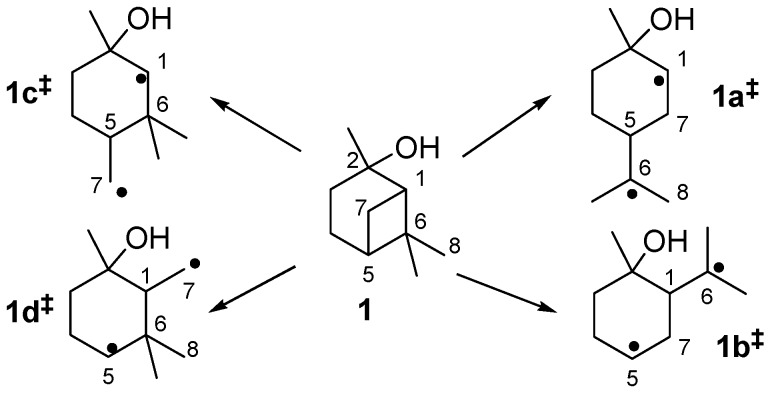
Possible radical intermediates **1i^‡^** in the thermal rearrangement of pinan-2-ol.

The thermal isomerization of **1** proceeds by a fragmentation of the cyclobutane ring. Therefore two possible mechanisms are discussed in literature, a concerted mechanism and a stepwise fragmentation [19,21,27,31,34]. According to Woodward-Hoffmann-rules the first mechanism is thermally forbidden, so it is generally accepted that the stepwise fragmentation is the most plausible way [35,36]. The mechanism starts with a breakage of a carbon-carbon bond either between C(1)-C(6), C(5)-C(7), C(1)-C(7), or C(5)-C(6) leading to four different biradicals **1i^‡^** (Scheme 4). In biradicals **1c^‡^** and **1d^‡^** the radicals are located on primary or secondary carbon atoms and are therefore less stable than tertiary isopropyl radicals in **1a^‡^** and **1b^‡^**. This leads to the conclusion that **1a^‡^** and **1b^‡^** are likely to be the preferred intermediates. However, **7** and its consecutive products, which would be generated from **1b^‡^**, were not found in the product mixture, although some authors have reported the formation of **10** during pyrolysis of **1** [20,21]. Thus, it seems that **1a^‡^** is the most plausible biradical intermediate. Products originating from this intermediate are **2**, **6**, and **8**, which account for >90% of the overall products formed (see Figure 4). Furthermore, similar results were found by Kinzel *et al.* with **4**, the parent compound of **1** [37]. They used quantum chemical methods to calculate the energetically preferred transition state including conformation of the cyclohexane ring. The preference for products formed via biradicals of type **1a^‡^** is also manifested for the thermal isomerization of other compounds with similar structures to **1** [19], namely *cis*-**4** [27,38,39], isopinocampheol (**15**) [40,41,42], isopinocamphone (**16**) [40,43], or *endo*-chrysanthenyl acetate (**17**; Figure 1) [44]. Contrarily, thermal isomerization of *trans*-**4** leads to a product distribution which can only be explained by a preference of both routes starting with biradicals from type **1a^‡^** and **1b^‡^** [28,37]. Structural constraints made responsible for this behaviour which are less expressed in the *cis*- and the *trans*-forms of **1**. Thus, no considerable effect on the radical stability and thus the product distribution was found (Figure 3).

Biradical **1a^‡^** leads to the formation of **2** and **6**, whereas **2** is formed by homolytic bond cleavage followed by intramolecular radical recombination. On the other hand, **6** is formed via sigmatropic [1,5]*H*-shift in which the hydrogen migrates from C(8) to C(1). The formation of **2** can be compared with conversion of β-pinene (**14**; see Figure 1) to myrcene. Formation of **6** proceeds similar to the formation of **12** from **3** or **13**.

A possible explanation for slight differences in reactivity and selectivity concerning the formation of **2** could be different activation energies and transition state geometries for the processes starting with *cis-* or *trans*-**1**. It is assumed that steric differences in *cis*- and *trans*-**1** have an influence on those parameters as shown for the hydrocarbon system [37]. As a result of that, *trans*-**1** is able to develop interaction with protons (from the *gem*-dimethylmethylene unit in the molecule) causing it to be more stable than *cis*-**1**. Furthermore, there is a weakening of the C(1)-C(6) bond of the pinane ring in *cis*-**1** by steric repulsion of the CH_3_-groups in the same direction. Also it has been found that the *cis*-isomer has a higher ring strain energy [14,21,33,45]. All these factors together make *trans*-**1** to a more stable molecule compared with *cis*-**1**, causing the higher on-set temperature for its conversion shown in Figure 3.

### 2.4. Mechanism for the Rearrangement of Linalool to Plinols

Compounds **8a**–**d** are formed via a concerted intramolecular (3,4)-ene reaction (Scheme 3), like many 1,6-dienes with hydrogen in α-position of any of the two double bonds [19,33,46]. Other examples are the cyclization of myrcene to iridane-1(6),8-diene [18] and the rearrangement of 7-methylocta-1,6-dien-3-one (from nopinone) to 2-methyl-3-isopropenylcyclopentanone [32,43,47]. The hybridization of C(2) is important for the number of cyclopentanols formed. The acyclic isomerization product **2** formed from **1** is sp^3^-hybridized in the respective position and thus generates four diastereomeric enantiomer pairs. If the generated compound has sp^2^-hybridization only two sets of enantiomers are formed (e.g., from **11**) [18,32]. Generally, the absolute configuration at C(3) of **2** remains unchanged during the pyrolysis [equal to C(1) in **1**]. Thus, by application of optical pure starting material [in the present case (*R*)-**2**] it is possible to assign the absolute configuration at C(1) in compounds **8**. The configurations of C(2) and C(3) in **8a**–**d** [and, therefore, also their relative configuration to C(1) with unchanged absolute configuration] are attributed to the different presumptive transition states. Generation of **8a**–**d** from **1** yielded rather the respective enantiomeric pairs than the optical pure cyclopentanols due to lower optical purity of **1** [ratio of (+)-*cis*-**1**:(−)-*cis*-**1** = 63:37] compared to (*R*)-**2**. Also **8c** and **8d** are the major products of linalool isomerization, **8a** and **8b** are the most stable ones due to the absence of repulsive 1,3-diaxial interactions of methyl substituents [18,27]. However, the preferred formation of **8c** and **8d** (Figure 8) with similar orientation of the alkyl substituents at C(2) and C(3) can be explained by the restrictive cyclic transition state shown in Scheme 3. DFT-calculation on the B3LYP/6-31G* level of theory for ene-cyclization of 7-methylocta-1,6-diene and 7-methylocta-1,6-dien-3-one proved this behavior [48].

### 2.5. Thermal Behavior of Pinan-2-ol in the Presence of Additives

As shown in Scheme 2 pyrolysis of **1** can lead to dehydration products **11** and **12**. To suppress these side reactions and increase the selectivity *S***_2_** and yield of linalool *Y***_2_**, experiments with five different additives added to the feed stream were carried out using the similar experimental condition as before. Table 2 and Table 3 summarize the results for pyrolysis at 450 and 525 °C, respectively. Small amounts of water, pyridine, or dimethylamine have only small influences on *Y***_2_**, *Y***_8_**, and conversion of **1** (*X***_1_**). The effect is more pronounced at higher temperatures (Table 3), since at low temperature the differences are within the experimental error. For the selectivity values (*S***_2_**) an increase from 41% (without additive) to 47% (dimethylamine or water as additive) was observed. This results in an increase of the yield of **2** by suppressing the consecutive reaction to **8**, since the overall selectivity for the pathway I (Scheme 2; *S***_p2_**) remains constant. In contrast to the literature the presence of additives rather influences the cyclization pathway from **2** to **8** than suppress dehydration. Semikolov *et al.* used various amounts of pyridine (0–16 mol%; normally 2 mol%) to suppress dehydration [14]. However, they have used a different apparatus with a “monolith composite catalyst” placed inside the reactor which could be an explanation related to different results, e.g., they observed even higher selectivity.

Addition of scavengers like aniline or toluene did not change conversion, yield, or selectivity significantly. In fact differences were even smaller than with the basic additives discussed before. But it is positive to note that no further side- or by-products were formed or observed. The fact that those radical scavengers did not show any influence can be explained by: (i) the short half live time of the biradical intermediates and (ii) the low probability of contact of two molecules compared to the large inert gas volume (*n***_1_**:*n*(N_2_) >> 1:1,000).

## 3. Experimental

### 3.1. Chemicals

Several lots of commercial-grade pinan-2-ol (**1**) were obtained from ChemSampCo (Trenton, NJ, USA; delivered via Chemie Brunschwig AG, Basel, Switzerland), with the following analytical data: 85–90 wt% *cis*-**1**, *cis*-**1**:*trans*-**1** > 99.5:0.5, (+)-*cis*-**1**:(−)-*cis*-**1** = 63:37 to 69:31, α-pinene (**3**) < 1.5%, β-pinene (**14**) < 0.5%. (*R*)-Linalool (**2**, ca. 95%) was purchased from Sigma-Aldrich (Basel, Switzerland). Both compounds were used without further purification. Purity was ascertained by capillary gas chromatography.

### 3.2. Pyrolysis Apparatus

Pyrolysis was carried out in an electrically heated quartz tube of 500 mm length and with a pyrolysis zone of 200 mm, using the apparatus reported in literature [32]. Temperatures (*T*) were regulated with thermocouples and in addition the actual *T* was measured inside the reactor. In all experiments, oxygen-free nitrogen with a purity of 99.999% was used as the carrier gas, unless described otherwise. Calculations of the average residence time τ were accomplished according to Equation (4).

For studying the influence of the surface-to-volume-ratio (*S/V*) the quartz tube inside the reactor was changed (Table 1). If not stated otherwise experimental set-up with insert was used. Therefore, the actual reactor geometry was similar to a hollow cylinder. Calculated reactor volumes (*V_R_*), *S/V*, and *τ* were based on a reaction zone with of 200 mm length. Within this zone the axial *T*-gradient in flow-direction was <10 K. Pyrolysis experiments used for calculation of rate constants were individually carried out at least two times. For further calculation all experimental results were considered.

### 3.3. Pyrolysis Experiments

The investigated monoterpenoids were pyrolyzed in a temperature range of 350–600 °C. Before pyrolysis **1** was dissolved in *n*-heptane while **2** was used undiluted. Furthermore, different pinan-2-ol solutions were prepared; each containing different additives at different concentration levels (Table 4).

The starting material (30 μL) was introduced on a quartz ladle into the top part of the pyrolysis apparatus using a glass syringe (50 μL). The starting material was carried along with the nitrogen stream into the reactor. Vaporization of the starting material was supported by heating the ladle to 200 °C with a hot blast. Pyrolysis products were collected in a cold trap (liquid nitrogen) and were dissolved in ethyl acetate (1.5 mL). The liquid products obtained were analyzed by FID-GC and GC-MS.

### 3.4. Analysis of the Pyrolysis Mixtures

Analyses of the dissolved (AcOEt) pyrolysis mixtures were carried out with GC-FID (Agilent Technologies 7890A) and GC-MSD (Agilent Technologies 6890N). Products were identified by comparison of either retention times and/or mass spectra of pure reference compounds. Analytical conditions for pyrolysis experiments with pinan-2-ol (**1**; GC-FID): HP 1, 30 m·0.32 mm·0.25 μm, 10 psi H_2_; program: 35 °C (hold 1 min), 4 Kmin^−1^ up to 80 °C, 4.5 K min^−1^ up to 90 °C, 35 K min^−1^ up to 280 °C (hold 3 min); injector temperature: 250 °C; detector temperature: 280 °C. Analytical conditions for pyrolysis experiments with linalool (**2**; GC-FID): HP 5, 30 m·0.32 mm·0.25 μm, 5 psi H_2_; program: 35 °C (hold 1 min), 4 K min^−1^ up to 80 °C, 4.5 K min^−1^ up to 90 °C, 35 K min^−1^ up to 280 °C (hold 3 min); injector temperature: 250 °C; detector temperature: 280 °C. Analytical conditions for GC-MSD: HP 5, 30 m·0.32 mm·0.25 μm, 7 psi He; program: 55 °C (hold 1 min), 5 K min^−1^ up to 150 °C, 20 K min^−1^ up to 280 °C (hold 5 min); injector temperature: 280 °C, EI (70 eV).

For the calculation of yields, conversions, and selectivities area percentages from the chromatograms have been used. Since the molecular formulas are similar (except dehydration products) no correction with respect for different FID-sensitivity was necessary. Due to the low concentration of the dehydration products and the fact that correction factors had a negligible effect on the reaction mixture composition respective corrections were not taken into account.

## 4. Conclusions

In summary, the thermal behavior of pinan-2-ol (**1**) and linalool (**2**) was investigated. The thermal isomerization of **1** proceeds via a biradical intermediate leading to acyclic **2** as main product and monocyclic β-terpineol (**6**) as major primary by-products (Scheme 1). Furthermore, **2** underwent *ene*-type cyclization reactions leading to cyclopentanols **8**. Additionally, dehydration reaction could arise forming monoterpenes myrcene (**11**) or limonene (**12**). The number of consecutive products formed depends on the reaction conditions. Short residence times and high temperatures lead to high selectivity and yield of **2** and small *S/V* increases those values, but at temperatures higher than 500 °C consecutive reactions are preferred, yielding products **8**. A change of the carrier gas to argon causes no perceptible difference in yield, selectivity, or conversion. With the use of basic additives like dimethylamine, the selectivity for **2** could be increased, while the presence of radical scavengers has no impact. The major function of the bases is the blockage of acidic centers at the surface of the reactor wall and thus, inhibition of dehydration to hydrocarbons. The ineffectiveness of radical scavengers can be traced back to the large mean free path length and the high thinning rate due to operation in the gas phase and the utilization of oxygen-free nitrogen as carrier gas, respectively.

## Figures and Tables

**Table 1:** Reactor properties.

Reactor geometry ^a^	Without insert cylinder	With hollow insert cylinder ^b^
*d*_reactor_ [mm]	15.2	15.2
*d*_insert_ [mm]	-	9.1
*V*_reactor_ [cm^3^]	36.1	23.0
*S*_reactor_ [cm^2^]	95.2	152.4
*S*_cross-section_ [cm^2^]	1.80	1.15
*S*/*V*-ratio [cm^−1^]	2.64	6.61

^a^ For details see [30] ^b^ If not indicated otherwise this set-up is used for the experiments;.

**Table 2:** Conversion *X***_1_**, selectivity *S***_2_**, yields *Y***_2_**, *Y***_8_**, and selectivity for pathway I (*S***_p2_**; for definition see Figure 4) for the pyrolysis of **1** with different additives at *T* = 450 °C ^a^.

Additive	*n*_1_:*n*_solvent_:*n*_additive_	*X*_1_ [%]	*S*_2_ [%]	*Y*_2_ [%]	*Y*_8_ [%]	*S*_p2_ [%]
None		13	61	8	2	77
Dimethylamine	1:1:0.1	8	80	7	1	100
Pyridine	1:1:0.1	12	57	7	0	58
Water	1:1:0.1	9	79	7	1	89
Dimethylamine	1:1:0.5	8	78	7	0	78
Pyridine	1:1:0.5	9	74	7	1	89
Water	1:1:0.5	9	79	7	1	89
Aniline	1:1:0.5	12	56	6	0	50
Toluene	1:1:0.5	10	67	7	0	70

^a^ Reaction conditions: 30 µL **1**, solvent: *n*-heptane, carrier gas N_2_, flow rate: 0.8 L min^−1^, *τ*: 0.6–0.8 s.

**Table 3:** Conversion *X***_1_**, selectivity *S***_2_**, yields *Y***_2_**, *Y***_8_**, and selectivity for pathway I (*S***_p2_**; for definition see Figure 4) for the pyrolysis of **1** with different additives at *T* = 525 °C ^a^.

Additive	*n*_1_:*n*_solvent_:*n*_additive_	*X*_1_ [%]	*S*_2_ [%]	*Y*_2_ [%]	*Y*_8_ [%]	*S*_p2_ [%]
None		92	41	38	44	89
Dimethylamine	1:1:0.1	90	47	42	38	89
Pyridine	1:1:0.1	89	46	41	35	85
Water	1:1:0.1	91	47	42	39	89
Dimethylamine	1:1:0.5	88	46	40	34	84
Pyridine	1:1:0.5	91	44	40	41	89
Water	1:1:0.5	91	45	41	41	90
Aniline	1:1:0.5	90	45	40	39	88
Toluene	1:1:0.5	90	45	41	39	89

^a^ Reaction conditions: 30 µL **1**, solvent: *n*-heptane, carrier gas N_2_, flow rate: 0.8 L min^−1^, *τ*: 0.6–0.8 s.

**Table 4:** List of pyrolysis experiments (solvent: *n*-heptane).

Additive	*n*_1_:*n*_solvent_:*n*_additive_	*T* [°C]
without additive	1:1:0	350–600
Dimethylamine	1:1:0.1, 1:1:0.5	350–600
Pyridine	1:1:0.1, 1:1:0.5	350–600
Water	1:1:0.1, 1:1:0.5	350–600
Aniline	1:1:0.5	425–525
Toluene	1:1:0.5	425–525
